# Safety and effectiveness of avelumab in patients with Merkel cell carcinoma in general clinical practice in Japan: Post‐marketing surveillance

**DOI:** 10.1111/1346-8138.17096

**Published:** 2024-03-03

**Authors:** Hisashi Uhara, Yoshio Kiyohara, Taiki Isei, Kotaro Nagase, Anzu Kambe, Masashi Sato, Yutaro Tanaka, Naoya Yamazaki

**Affiliations:** ^1^ Sapporo Medical University School of Medicine Sapporo Hokkaido Japan; ^2^ Shizuoka Cancer Center Hospital and Research Institute Shizuoka Japan; ^3^ Osaka International Cancer Institution Osaka Japan; ^4^ Department of Dermatology Saga‐Ken Medical Centre Koseikan Saga Japan; ^5^ Merck Biopharma Co., Ltd. Tokyo Japan, an affiliate of Merck KGaA; ^6^ National Cancer Center Hospital Tokyo Japan

**Keywords:** avelumab, Merkel cell carcinoma, post‐marketing, safety, treatment effectiveness

## Abstract

Avelumab, a programmed cell death ligand 1 blocking antibody, was approved for its first indication in Japan in September 2017 to treat unresectable Merkel cell carcinoma (MCC). Given that the pivotal JAVELIN Merkel 200 study only included a few Japanese patients, this post‐marketing surveillance (PMS) evaluated the safety and effectiveness outcomes of patients with MCC who received avelumab in general clinical practice in Japan. This prospective, non‐comparative, multicenter PMS included data from all patients with unresectable MCC who received avelumab between November 22, 2017 (avelumab launch date) and October 31, 2019. The primary objective was to evaluate avelumab safety (i.e., adverse events [AEs], adverse drug reactions [ADRs], and ADRs of safety specifications). The secondary objective was to evaluate avelumab effectiveness (i.e., objective response rate and overall survival [OS] rate). Seventy‐five evaluable patients were included, of whom 81.3% experienced AEs of any grade (57.3% experienced AEs of grade ≥ 3; 41.3% experienced AEs of grade 5) and 61.3% experienced ADRs (14.7% experienced ADRs of grade ≥ 3; no grade 5 ADRs were observed). The most common ADRs were pyrexia (18.7%), infusion related reaction (10.7%), and chills (6.7%). The most common ADRs of safety specifications were infusion reactions (any grade: *n* = 21 [28.0%]; grade 3 or 4: *n* = 3 [4.0%]), thyroid dysfunction (*n* = 7 [9.3%]), and hepatic function disorders (*n* = 4 [5.3%]). The median observation period was 51 weeks. An objective response was achieved by 34/75 patients (45.3%; complete response, 24.0%; partial response, 21.3%) and 6‐ and 12‐month OS rates were 77.7% and 59.6%, respectively. This PMS confirmed the clinical tolerability and effectiveness of avelumab in patients with MCC, with no new safety concerns. The risk–benefit profile of avelumab was comparable with that observed in clinical trials and remains favorable for use in general clinical practice in Japan.

## 1 INTRODUCTION

Merkel cell carcinoma (MCC) is a rare, aggressive, neuroendocrine skin cancer^1^, ^2^ and is the second most common cause of skin‐cancer‐related death after melanoma.^3^ The incidence of MCC varies geographically with the highest incidence reported in Australia (age‐adjusted incidence of 1.6 per 100 000) and a low incidence in Asia, relative to other countries.^1^, ^2^ Documented risk factors for the development of MCC include advancing age, ultraviolet (UV) light exposure, immunosuppression, and Merkel cell polyomavirus infection (MCPyV).^4^, ^5^ However, there are also notable geographical differences: in the Northern Hemisphere, the majority of MCCs (80%) are typically associated with MCPyV and a similar trend has been observed in Japan (68.9%), whereas the presence of MCPyV is reported in approximately 30% of Australian patients with MCC.^1^, ^2^ UV light‐mediated mutations account for the remaining cases.^1^, ^2^ Although a rare disease, the incidence of MCC is increasing globally.^2^, ^6^ This may partially be due to improvements in diagnostics and increases in disease awareness, aging populations, and UV light exposure.^6^

MCC often metastasizes early, and prognosis is poor for many patients because of a lack of effective and tolerable treatments, particularly for advanced disease stages.^5^, ^7^ Until 2017, no agents were specifically approved for metastatic/unresectable MCC. Although MCC is chemosensitive, the response duration to chemotherapy in metastatic MCC is generally low with a minimal survival benefit.^4^, ^8^ Avelumab, a programmed cell death ligand 1 (PD‐L1) blocking antibody, was initially granted accelerated approval for metastatic MCC in the United States in March 2017^9^. It has since been approved in numerous countries, including Japan, where it was approved in September 2017 for curatively unresectable MCC.^10^ Subsequently, in September 2023, avelumab was granted full approval in the United States for metastatic MCC in patients aged ≥12 years old, becoming the first and only immune checkpoint inhibitor with full approval for this indication in the United States.^11^ This approval was based on the results of the international phase 2 JAVELIN Merkel 200 study, which demonstrated the effectiveness of avelumab both in patients with chemotherapy‐refractory metastatic MCC (Study Part A)^12^ and as first‐line treatment in patients with metastatic MCC (Study Part B).^13^, ^14^ Since then, several retrospective studies have also published real‐world data on avelumab use in MCC as a first‐ or second‐line treatment, confirming findings from the JAVELIN Merkel 200 study.^4^, ^8^, ^15^, ^16^, ^17^

As only three Japanese patients were enrolled in the pivotal JAVELIN Merkel 200 study, post‐marketing surveillance (PMS) was required to confirm the safety and effectiveness of avelumab for its approval in Japan. The current PMS presents the results of first‐line treatment of avelumab in patients with MCC from an unbiased, all‐case surveillance.

## 2 MATERIALS AND METHODS

### 2.1 Study design and patient population

This prospective, non‐comparative, multicenter PMS was designed to evaluate the safety and effectiveness of avelumab in patients with MCC in general clinical practice in Japan. This PMS included data from all patients with unresectable MCC who started treatment with avelumab in Japan within a 23‐month time frame from its launch on November 22, 2017 to October 31, 2019.

All surveillance was conducted in accordance with the Japanese regulations of Good Post‐marketing Study Practice (GPSP).^18^ While approval from ethics committees/institutional review boards and informed patient consent were not mandated by the GPSP ordinance, the protocol was submitted to the participating institutions/investigators and written informed consent was obtained from the patients based on the requirements of each institution.

Since this PMS was designed as an all‐case surveillance, all patients with unresectable MCC who received at least one dose of avelumab were enrolled. Patient enrolment was centralized. The sponsor collected case report forms (CRFs) which investigators filled out after observing each patient for a maximum of 52 weeks after the first dose.

As MCC is a rare disease, the target number of patients was set as 48. This was in consideration of feasibility during the re‐examination period and the incidence of adverse drug reactions (ADRs) reported in an integrated analysis of three clinical trials of avelumab including the JAVELIN Merkel 200 study (data on file). It was initially assumed that patients would be enrolled over 5 years; however, the target number of patients was exceeded in September 2019.

### 2.2 Endpoints

The primary objective was to evaluate avelumab safety in general clinical practice in Japan. Safety endpoints included the incidence of adverse events (AEs)/ADRs and the incidence of ADRs of safety specifications, as described by the Japanese Risk Management Plan for avelumab. ADRs of safety specifications included (1) infusion reactions (Supplementary Methods); (2) immune‐related ADRs (i.e., interstitial lung disease [ILD], hepatic function disorders, colitis/severe diarrhea, thyroid dysfunction, adrenal insufficiency, type 1 diabetes mellitus, myocarditis, nerve disorders, renal disorders, and myositis/rhabdomyolysis); and (3) other important potential risks (i.e., encephalitis/meningitis, embryo/fetal toxicity, and the incidence of graft rejection or graft‐versus‐host disease in patients with an organ transplant history, including a history of hematopoietic stem cell transplantation). In this PMS, the severity of AEs was evaluated using the Common Terminology Criteria for Adverse Events (grades 1–5). Additionally, the investigators could classify an AE as serious if it resulted in death, was life‐threatening, required hospitalization or prolonged hospitalization, resulted in a persistent or significant disability, was a congenital anomaly or birth defect, or was otherwise considered as medically important. ADRs were defined as any AEs that were considered to be related to avelumab treatment.

The secondary objective was to evaluate avelumab effectiveness in general clinical practice. Effectiveness endpoints were objective response (i.e., best overall response, objective response rate [ORR]), and overall survival (OS), defined as time to all‐cause death. Objective response was categorized into five categories of best overall response (complete response [CR], partial response [PR], stable disease [SD], progressive disease [PD], and not evaluable [NE]), which was determined by investigators with reference to the Response Evaluation Criteria in Solid Tumors Guideline (version 1.1).^19^

### 2.3 Statistical analyses

Both the safety and effectiveness analysis populations included data from the ‘finalized CRF population’ whose CRFs had been collected and the data locked from the start of the PMS (November 22, 2017) until the date of database lock (September 22, 2021). For the safety analysis, data were presented as overall frequency and percentages, aggregated by system organ class and preferred term (PT), as well as by severity, seriousness, time of onset, and outcome. The rate and proportion of each best overall response (i.e., CR, PR, SD, PD, or NE) were calculated for the effectiveness analysis. For ORR (i.e., the best overall response of CR or PR) and disease control rate (DCR; i.e., the best overall response of CR, PR, or SD), the proportion of patients and Clopper–Pearson 95% confidence intervals (CI) were calculated. For OS, Kaplan–Meier estimates and associated statistics (e.g., survival rate and median survival time at each time point) and their 95% CIs were calculated.

## 3 RESULTS

### 3.1 Patients and treatment

**FIGURE 1:**
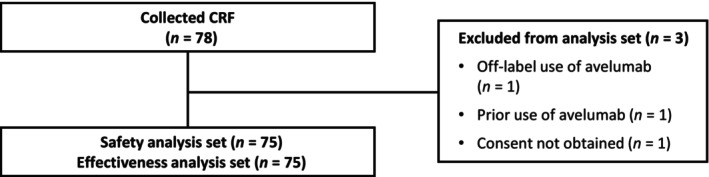
Study flowchart. CRF, case report form.

At database lock (September 22, 2021), CRFs were collected from 78 patients across 55 institutions. Of these patients, 75 patients across 53 institutions were included in the safety and effectiveness analyses. Patients were excluded for off‐label use of avelumab, prior use of avelumab, and not providing consent (*n* = 1 each; Figure 1).

At baseline, the median (range) patient age was 77 years (range, 42–95), the male‐to‐female ratio was similar (48.0% and 52.0%, respectively), and PD‐L1 status was unknown for the majority of patients (89.3%; Table 1; Table S1). Most patients (81.3%) had an Eastern Cooperative Oncology Group (ECOG) performance status (PS) of 0 or 1; 13 patients (17.3%) had ECOG PS 2 or 3 (which was an exclusion criterion for the JAVELIN Merkel 200 study), and no patient with ECOG PS 4 was reported. Avelumab treatment was initiated as first‐line therapy in 70/75 patients (93.3%) with MCC. The median number of doses was 11.0 (range, 1–27 doses), at a median dosage of 10.0 mg/kg (range, 5.9–11.4 mg/kg; Table S1). The approved 10 mg/kg dosage was commonly administered in general clinical practice. The median observation period was 51 weeks (range, 1.3–61.1 weeks). The primary reason for death among nine patients who discontinued avelumab treatment was disease progression (*n* = 8) and death caused by an AE of pneumocystis jirovecii pneumonia (*n* = 1), which was not considered to be related to avelumab.

**TABLE 1:** Baseline characteristics^a^

Characteristic	*N* = 75
Sex, *n* (%)	
Male	36 (48.0)
Female	39 (52.0)
Age, median (range), years	77.0 (42–95)
ECOG PS, *n* (%)	
0	36 (48.0)
1	25 (33.3)
2	7 (9.3)
3	6 (8.0)
4	0
Unknown	1 (1.3)
Location of underlying disease, *n* (%)	
Skin	67 (89.3)
Lymph node	2 (2.7)
Other	6 (8.0)
PD‐L1 status, *n* (%)	
Positive	1 (1.3)
Negative	7 (9.3)
Unknown	67 (89.3)
Comorbidities, *n* (%)	
Renal impairment	11 (14.7)
Hepatic impairment	8 (10.7)
ILD	4 (5.3)
Autoimmune disease	7 (9.3)
MCC treatment history, *n* (%)^b^ ^,^ ^c^	
Surgery	57 (76.0)
Radiation	47 (62.7)
Chemotherapy	7 (9.3)

Abbreviations: ECOG PS, Eastern Cooperative Oncology Group performance status; ILD, interstitial lung disease; MCC, Merkel cell carcinoma; PD‐L1, programmed death‐ligand 1.

^a^ Further baseline characteristics and status of avelumab treatment are shown in Table S1).

^b^ Patients could be counted in more than one category.

^c^ Of seven patients with a history of chemotherapy use, two received postoperative adjuvant therapy. Since >6 months had elapsed between the end of chemotherapy and the use of avelumab, these two patients were considered to have received avelumab as first‐line treatment for unresectable MCC, and the other five patients received avelumab as second‐line treatment.

#### 3.1.1 Treatment discontinuation

Overall, 50 patients (66.7%) discontinued avelumab treatment, with the most common reasons reported by the investigators being disease progression (33.3%), death (12.0%), and AEs (12.0%). The PTs of the AEs reported as reasons for treatment discontinuation by investigators were infusion related reaction, radiation pneumonitis, type 1 diabetes mellitus, oral candidiasis, renal impairment, portal vein thrombosis, cerebellar ataxia, decreased white blood cell count, decreased neutrophil count, and myositis (*n* = 1 each, one patient had both decreased white blood cell and neutrophil counts). Of these, oral candidiasis was non‐serious and grade 2 in severity, and the other events were all serious and of grade 3 severity or higher. Regarding the outcomes of these AEs, type 1 diabetes mellitus and renal impairment remained unresolved, and the other events were resolving or resolved.

### 3.2 Safety

In the safety analysis set, 61/75 patients (81.3%) experienced an AE of any grade, and 43/75 patients (57.3%) experienced an AE of grade ≥ 3 after initiating avelumab treatment. In terms of ADRs, 46/75 patients (61.3%) experienced an ADR of any grade, and 11/75 patients (14.7%) experienced an ADR of grade ≥ 3. Nine patients (12.0%) developed a grade 3 ADR, and three patients (4.0%) developed a grade 4 ADR (one patient had both grade 3 and 4 ADRs); no patients developed grade 5 ADRs (Table 2). The most common ADRs (i.e., those occurring in >5% of patients) of any grade were pyrexia (18.7%), infusion related reaction (10.7%), and chills (6.7%); Table S2 shows details of ADRs reported in this PMS.

**TABLE 2:** Adverse drug reactions in the safety analysis set (*N* = 75).

*n* (%)	Worst Grade				Total
	1	2	3	4	
Any ADR	32 (42.7)	17 (22.7)	9 (12.0)	3 (4.0)	46 (61.3)
ADRs occurring in ≥2% of patients					
Pyrexia	11 (14.7)	2 (2.7)	1 (1.3)	0	14 (18.7)
Infusion related reaction	3 (4.0)	3 (4.0)	1 (1.3)	1 (1.3)	8 (10.7)
Chills	5 (6.7)	0	0	0	5 (6.7)
Diarrhea	2 (2.7)	1 (1.3)	0	0	3 (4.0)
Pruritus	2 (2.7)	1 (1.3)	0	0	3 (4.0)
Adrenal insufficiency	1 (1.3)	1 (1.3)	0	0	2 (2.7)
ALT increased	2 (2.7)	0	0	0	2 (2.7)
AST increased	2 (2.7)	0	0	0	2 (2.7)
Drug eruption	1 (1.3)	1 (1.3)	0	0	2 (2.7)
Hyponatremia	0	1 (1.3)	1 (1.3)	0	2 (2.7)
Hypothyroidism	2 (2.7)	0	0	0	2 (2.7)
ILD	2 (2.7)	0	0	0	2 (2.7)
Thyroiditis	1 (1.3)	1 (1.3)	0	0	2 (2.7)

Abbreviations: ADR, adverse drug reaction; ALT, alanine aminotransferase; AST, aspartate aminotransferase; ILD, interstitial lung disease.

#### 3.2.1 Infusion reactions

In the safety analysis set, 21/75 patients (28.0%) developed at least one ADR that was classified as an infusion reaction (Table S3), including pyrexia (*n* = 10 [13.3%]), infusion related reaction (*n* = 8 [10.7%]), and chills (*n* = 5 [6.7%]). Other infusion reactions included atrial fibrillation, increased C‐reactive protein, dyspnea, and increased procalcitonin (each *n* = 1 [1.3%]). Three patients experienced grade ≥ 3 infusion reactions (one each experienced grade 3 and 4 infusion related reactions, and one experienced grade 3 pyrexia) (Figure S1). Serious infusion reactions were reported in five patients (6.7%; Table S3); all grade ≥ 3 infusion reactions were serious. Among grade 1 or 2 infusion reactions, increased C‐reactive protein, increased procalcitonin, pyrexia, and infusion related reactions (each *n* = 1 [1.3%], with overlapping patients) were reported as serious. These events were classified as serious ADRs despite being grade 1 or 2 due to the requirement for hospital admission/prolonged hospitalization. All reported infusion reactions were resolved. Of the patients who developed at least one infusion reaction (*n* = 21), four patients developed additional infusion reaction events on the first and second doses (*n* = 2), on the seventh and ninth doses (*n* = 1), and on the third, sixth, seventh, and eleventh doses (*n* = 1). All four patients had a recurrence of the same event.

**FIGURE 2:**
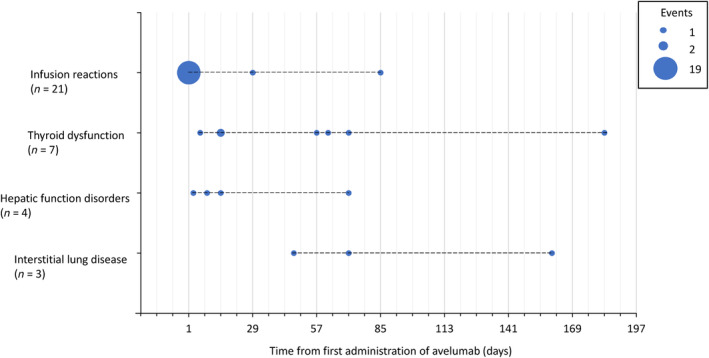
Time to first onset of adverse drug reactions of safety specifications. Among the safety specifications, the following adverse reactions were observed in <3 patients. The number of days from the first administration of avelumab was as follows: nerve disorders (*n* = 2, days 85 and 116); adrenal insufficiency (*n* = 2, days 14 and 121); colitis/severe diarrhea (*n* = 1, day 170); type 1 diabetes mellitus (*n* = 1, day 141); myositis/rhabdomyolysis (*n* = 1, day 29); and renal disorders (*n* = 1, day 92).

The time to first infusion reaction onset is shown in Figure 2. Nineteen patients experienced their first infusion reaction on the day of the first avelumab administration; the remaining two patients experienced their first infusion reaction on the days of the third and seventh administrations, respectively. All grade ≥ 3 infusion reactions occurred on the day of the first avelumab administration. Fourteen out of 21 patients experienced their first infusion reaction within 1 h of avelumab administration (Table S4). Of the 75 patients, 73 received pre‐medication at the first avelumab administration to prevent infusion reactions. The incidence of infusion reactions did not significantly vary by the type of pre‐medication used. Coincidentally, no infusion reactions were observed in the two patients who did not receive pre‐medication (Tables S1 and S5).

#### 3.2.2 Other ADRs of safety specifications

The number of patients experiencing ADRs of safety specifications other than infusion reactions included seven with thyroid dysfunction (9.3%), four with hepatic function disorders (5.3%), three with ILD (4.0%), two patients each with nerve disorders and adrenal insufficiency (2.7%), and one patient each with colitis/severe diarrhea, type 1 diabetes mellitus, myositis/rhabdomyolysis, and renal disorders (1.3%; Table S3). None of the patients developed myocarditis, encephalitis/meningitis, embryo/fetal toxicity, or graft rejection/graft‐versus‐host disease.

The time to first onset of ADRs other than infusion reactions was as follows: six out of seven patients (85.7%) experienced the first onset of thyroid dysfunction within 84 days of treatment initiation. Three out of four patients (75.0%) experienced the first onset of hepatic function disorders within 28 days of treatment initiation. Three patients experienced the first onset of ILD after 28 days of treatment initiation. No consistent pattern of onset was seen in the ADRs of other safety specifications (Figure 2).

ADRs were mostly resolved or resolving by the end of the observation period; however, four ADRs of safety specifications remained unresolved or resolved with sequelae. Adrenal insufficiency remained unresolved after 253 days (*n* = 1); type 1 diabetes mellitus remained unresolved after 288 days (*n* = 1); renal disorder remained unresolved after 503 days (*n* = 1); and thyroiditis resolved after 29 days in one patient who experienced sequelae.

Other than infusion reactions, grade ≥ 3 ADRs of safety specifications were observed in four patients. One patient developed grade 4 ILD 71 days after treatment initiation (PT: radiation pneumonitis; resolved 18 days after onset with treatment discontinuation), one patient developed grade 3 myositis/rhabdomyolysis 29 days after treatment initiation (PT: myositis; resolved 56 days after onset with treatment discontinuation), one patient developed grade 3 type 1 diabetes mellitus 141 days after treatment initiation (PT: type 1 diabetes mellitus; remained unresolved at 288 days after onset despite treatment discontinuation), and one patient developed grade 3 nerve disorders 116 days after treatment initiation (PT: cerebellar ataxia; was resolving by 252 days after onset with treatment discontinuation).

All grade ≥ 3 ADRs were serious; among grade 1 or 2 ADRs, two were serious (Table S3). One patient developed grade 2 thyroid dysfunction 61 days after treatment initiation (PT: hyperthyroidism; resolved 113 days after onset with treatment suspension; the reason was hospital admission/prolonged hospitalization), and another patient developed grade 2 thyroid dysfunction 57 days after treatment initiation (PT: thyroiditis; remained unresolved 29 days after onset despite treatment suspension; the reason for seriousness was that the investigator assessed unresolved thyroiditis as medically important).

### 3.3 Effectiveness

**FIGURE 3:**
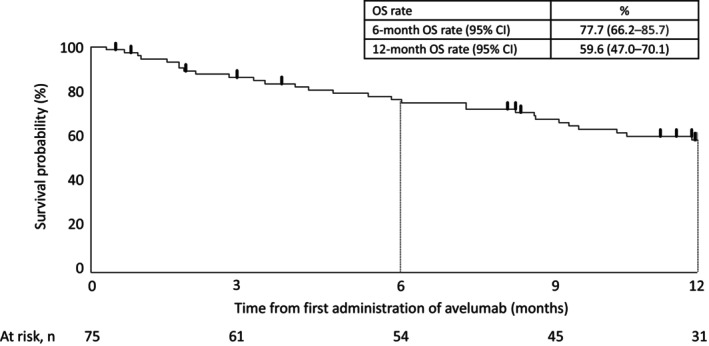
Kaplan–Meier curve of overall survival in the effectiveness analysis set (*N* = 75). CI, confidence interval; OS, overall survival.

The best overall response within 52 weeks of the first avelumab dose in the effectiveness analysis set was CR in 18/75 patients (24.0%), PR in 16 patients (21.3%), SD in eight patients (10.7%), and PD in 25 patients (33.3%). The ORR was 45.3% (34/75 patients), and the DCR was 56.0% (42/75 patients) (Table S6). The treatment response and duration for each patient is shown as a swimmer plot in Figure S2. The 6‐ and 12‐month OS rates were 77.7% (95% CI 66.2–85.7) and 59.6% (95% CI 47.0–70.1), respectively (Figure 3). Of the 50 patients who discontinued avelumab, six (8%) had a CR (*n* = 4, 5.3%) or PR (*n* = 2, 2.7%) after discontinuation.

## 4 DISCUSSION

To the best of our knowledge, this PMS is the first comprehensive dataset of avelumab for patients with unresectable MCC treated in general clinical practice in Japan. As an all‐case surveillance, it included all patients treated with avelumab from its launch on November 22, 2017 to October 31, 2019. The results of this PMS confirm that the clinical tolerability, safety, and effectiveness of avelumab in these patients in general clinical practice is comparable with the safety and efficacy profile observed in the JAVELIN Merkel 200 clinical trial.^12^ The incidence of ADRs of any grade in this PMS (61.3%) aligned with treatment‐related adverse events (TRAEs) of any grade observed in Part A^12^ and Part B^13^ of the JAVELIN Merkel 200 study (70.5% and 81.0%, respectively). The incidence of grade ≥ 3 ADRs in this PMS (14.7%) was higher than the incidence of grade ≥ 3 TRAEs in Part A (4.5%) of the JAVELIN Merkel 200 study,^12^ but similar to Part B (17.2%),^12^ i.e., severe ADRs observed in this PMS were within the expected range. Although this PMS involved a broader patient population than clinical trials, the incidence of ADRs in this PMS was generally consistent with the rates observed in the JAVELIN Merkel 200 study. This outcome indicates that avelumab is well tolerated across a wide patient population in general clinical practice in Japan.

The most common ADR of safety specifications in this PMS was infusion reactions, which occurred in 28.0% of patients and was of grade ≥ 3 severity in 4.0% of patients. It is worth mentioning that all infusion reactions occurred on the same day as avelumab administration, especially following the first dose (in 90.5% of patients). More specifically, 66.7% of patients developed infusion reactions within 1 h of initiation of avelumab administration, while 28.5% of patients developed infusion reactions >1 h initiation of initiating avelumab administration (details on the onset of infusion reaction in one patient were unknown). Based on these results, it is recommended that patients be carefully observed for several hours after administration of avelumab, particularly the first dose.

To prevent infusion reactions, patients treated with avelumab must be premedicated with antihistamines or antipyretic analgesics, according to the Japanese electronic package insert. The results from this PMS indicate that premedication was effectively managed in general clinical practice in Japan. These results also suggest that premedication may decrease, but not eliminate, the risk of infusion reactions.

Most ADRs of safety specifications (including grade ≥ 3 events) were resolved or were resolving by the end of the observation period excluding four cases, which means that they were manageable with appropriate treatment. Overall, most of the ADRs seen in this PMS were also observed as TRAEs in the JAVELIN Merkel 200 study and are already mentioned in the electronic package insert,^20^ as well as other related documents used in Japan.

The ORR in this PMS (45.3%) was comparable to Part A of the JAVELIN Merkel 200 study (31.8% [median follow‐up, 10.4 months; range, 8.6–13.1 months])^12^ and Part B (39.7% [median follow‐up, 21.2 months; range, 14.9–36.6 months]).^13^ The 6‐ and 12‐month OS rates in this PMS (77.7% and 59.6% respectively) were also similar to those observed in Part A (6‐month OS, 69%)^12^ and Part B (6‐month OS, 75%; 12‐month OS, 60%)^13^ of the JAVELIN Merkel 200 study. Although avelumab effectiveness in general clinical practice should not be compared directly to clinical trial results, the similarities between the ORR and OS suggest that the avelumab treatment benefit observed in the JAVELIN Merkel 200 study is mirrored in general clinical practice in Japan.

This PMS did not investigate re‐treatment after discontinuation, so the impact of avelumab rechallenge and chemotherapy after avelumab is unknown. Of the 25 patients experiencing PD, 14 patients continued treatment with avelumab for at least 1 month beyond disease progression. Notably, patients with advanced solid tumors treated with immunotherapy can develop atypical response patterns, whereby an initial PD response later results in tumor regression or prolonged disease stabilization.^21^, ^22^

This PMS also provides insights into patient demographics and other epidemiological information in Japan. The number of patients enrolled in this PMS was higher than expected and recruitment occurred faster than planned. This may be due to increased disease awareness following the approval of avelumab. MCC is known to differ by geographic location and by racial and ethnic groups.^23^ Because considerable differences in most skin cancers have been observed between Caucasian and Asian populations, positive clinical trial results obtained in Western countries need to be confirmed in the Japanese population.^23^ In contrast to prior reports on treatment outcomes from real‐world studies conducted in Europe^24^ and the United States,^25^ where a higher prevalence of males was observed, the current PMS demonstrated a similar proportion of females and males. The median age recorded in this PMS (77.0 years) closely aligned with that reported in the United States study mentioned above (75.8 years),^25^ while the European study cohort exhibited a slightly younger median age (67.5 years).^24^ Such information could aid in the better understanding of patient profiles to determine an age demographic of patients who may most benefit from avelumab. The difference in the sex ratio in this PMS compared with Europe or the United States could potentially be attributed to variations in the proportion of UV light exposure or presence of MCPyV, given their established roles in MCC. However, it is expected that more real‐world data will be accumulated to better understand these demographic differences.

Our PMS has some limitations. First, this was a non‐interventional, observational study with no control group, so it is difficult to clarify whether the obtained results were caused by exposure to avelumab. Second, the data were collected through a CRF filled out by the investigator and not reviewed by an independent data monitoring committee. Third, source data verification against medical records was not performed because this was not required by the GPSP ordinance. Results may be biased due to investigators' assessments. Additionally, caution is required when evaluating the OS data because of the limited observation period for each patient. Finally, we did not collect data on MCPyV status and only had limited data on PD‐L1 status in the patients in this PMS.

## 5 CONCLUSIONS

This PMS confirmed the clinical tolerability of avelumab in patients with unresectable MCC in Japan with no new safety concerns. The effectiveness of avelumab was also broadly similar to that reported in the JAVELIN Merkel 200 study. These results indicate that the risk–benefit profile of avelumab is comparable with that observed in the JAVELIN Merkel 200 study and remains favorable when used in general clinical practice.

## FUNDING INFORMATION

The study was funded by Merck (CrossRef Funder ID: 10.13039/100009945) and was previously conducted under an alliance between Merck and Pfizer.

## CONFLICT OF INTEREST STATEMENT

Hisashi Uhara received grants from Ono Pharmaceutical Co., Ltd., Bristol Myers Squibb, Novartis K.K., Minophagen Pharmaceutical Co., Ltd., Regeneron Pharmaceuticals, AbbVie G.K., Sato Pharmaceutical Co., Ltd., Japan Blood Products Organization, Syneron Candela K.K., Taiho Pharmaceutical Co., Ltd., Kaken Pharmaceutical Co., Sanofi, Eli Lilly Japan, Maruho, Tsumura, Daiichi Sankyo, Torii Pharmaceutical, Kyowa Kirin, Eisai, Pola Pharma, Nihon Pharmaceutical, Teikoku Seiyaku, Sun Pharma, and Public Trust Ono Cancer Research Grant Fund; he received consulting fees from Ono Pharmaceutical Co., Ltd., Bristol Myers Squibb, Novartis Pharma K.K., and Merck Biopharma Co., Ltd., Tokyo, Japan, an affiliate of Merck KGaA; he received honoraria for lectures from Nikkei Radio Broadcasting Co., AbbVie G.K., Eisai Inc., Ono Pharmaceutical Co., Ltd., Bristol Myers Squibb, Kaken Pharmaceutical Co., Ltd, Kyowa Kirin Co., Ltd., Convatec Japan K.K., Sanofi, Torii Pharmaceuticals, Eli Lilly Japan, Japan Pharmaceuticals, Novartis K.K., Maruho, Merck Biopharma Co., Ltd., Tokyo, Japan, an affiliate of Merck KGaA, Medical Review Corporation, Janssen Pharma, Chugai Pharmaceutical, Leo Pharma, Syneron Candela K.K., Toray Industries, Inc. Celgene Corporation, and MSD; he participated on the advisory board of Ono Pharmaceutical Co., Ltd., Bristol Myers Squibb, Novartis K.K, Merck Biopharma Co., Ltd., and Tokyo, Japan, an affiliate of Merck KGaA; he is a chairman of NPO Young Education in Skin Oncology, a vice chairman of Japanese Skin Cancer Society. Yoshio Kiyohara received grants from Bristol Myers Squibb, and Sanofi; he received honoraria for lectures from Novartis, AstraZeneca, Ono Pharmaceutical Co., Ltd., Janssen Pharma, Maruho, Bristol Myers Squibb, and Merck Biopharma Co., Ltd., Tokyo, Japan, an affiliate of Merck KGaA; and participated on advisory boards of Otsuka Pharmaceutical, and Merck Biopharma Co., Ltd., Tokyo, Japan, an affiliate of Merck KGaA. Taiki Isei received honoraria for lectures from Novartis, Ono Pharmaceutical Co., Ltd., Maruho, MSD K.K., Bristol Myers Squibb, DAIICHI SANKYO Co., LTD., Sun Pharmaceutical Industries Ltd., Kyowa Hakko Kirin Co., Ltd., and Merck Biopharma Co., Ltd., Tokyo, Japan, an affiliate of Merck KGaA; and participated on advisory boards of Novartis, Ono Pharmaceutical Co., Ltd., MSD K.K., Bristol Myers Squibb, and Merck Biopharma Co., Ltd., Tokyo, Japan, an affiliate of Merck KGaA. Anzu Kambe, Yutaro Tanaka, and Masashi Sato are employees of Merck Biopharma Co., Ltd., Tokyo, Japan, an affiliate of Merck KGaA. Naoya Yamazaki received royalties or license fees from Ono Pharmaceutical Co., Ltd., Amgen Inc., Bristol Myers Squibb, and Novartis Pharma K.K. and received honoraria for lectures from Ono Pharmaceutical Co., Ltd., MSD K.K., Bristol Myers Squibb, Maruho Co., Ltd., and Novartis Pharma K.K. Kotaro Nagase has no conflicts of interest/financial disclosure to declare.

## Supporting information


Figure S1.

Figure S2.

Table S1.

Table S2.

Table S3.

Table S4.

Table S5.

Table S6.

Data S1.

## Data Availability

Any requests for data by qualified scientific and medical researchers for legitimate research purposes will be subject to Merck's (CrossRef Funder ID: 10.13039/100009945) Data Sharing Policy. All requests should be submitted in writing to Merck's data sharing portal (https://www.merckgroup.com/en/research/our‐approach‐to‐research‐and‐development/healthcare/clinical‐trials/commitment‐responsible‐data‐sharing.html). When Merck has a co‐research, co‐development, co‐marketing or co‐promotion agreement, or when the product has been out licensed, the responsibility for disclosure might be dependent on the agreement between parties. Under these circumstances, Merck will endeavor to gain agreement to share data in response to requests.
