# Supplementary material for: Safety and effectiveness of avelumab in patients with Merkel cell carcinoma in general clinical practice in Japan: Post‐marketing surveillance
Source: J Dermatol. 2024 Mar 3;51(4):475–83. doi: 10.1111/1346-8138.17096 (PMC11484154; doi:10.1111/1346-8138.17096)

**SUPPLEMENTARY FIGURE S1** Timing of each patient’s first experience of an infusion reaction, by grade. At total of 21 patients developed at least one infusion reaction.


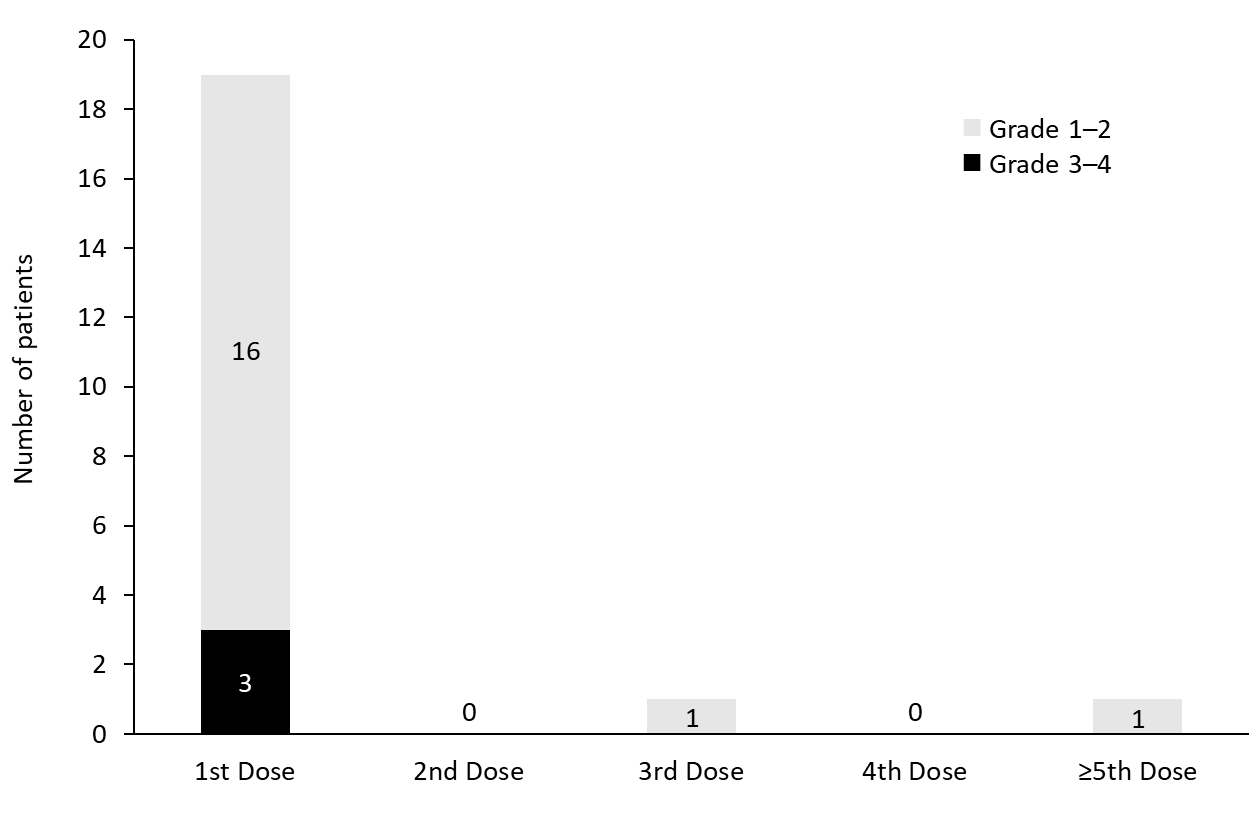

Supplement: Supplementary file 1 — Figure S1. [file JDE-51--s005.docx]
