# Supplementary material for: Safety and effectiveness of avelumab in patients with Merkel cell carcinoma in general clinical practice in Japan: Post‐marketing surveillance
Source: J Dermatol. 2024 Mar 3;51(4):475–83. doi: 10.1111/1346-8138.17096 (PMC11484154; doi:10.1111/1346-8138.17096)

**SUPPLEMENTARY FIGURE S2** Duration of avelumab treatment and duration of response for each evaluable patient (N=75) is represented, with the bar end representing the duration of 14 days added to the last dose date. Stratified by immune-related side effects after the first dose of avelumab; 1L, patients receiving first-line avelumab; 2L, patients receiving second-line avelumab; CR, complete response; irADR; immune-related adverse drug reaction; PR, partial response; PD, progressive disease.


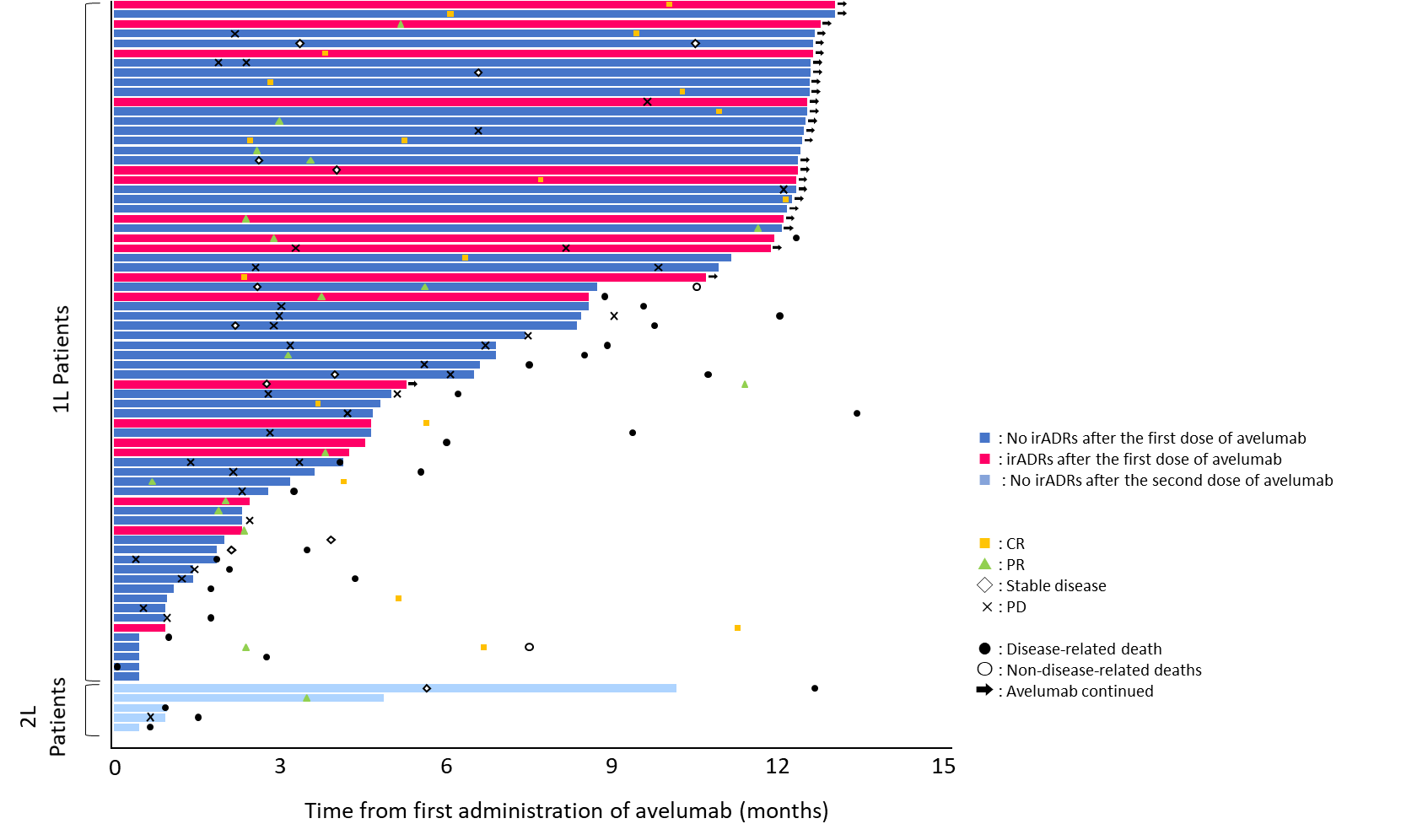

Supplement: Supplementary file 2 — Figure S2. [file JDE-51--s001.docx]
