# Supplementary material for: Safety and effectiveness of avelumab in patients with Merkel cell carcinoma in general clinical practice in Japan: Post‐marketing surveillance
Source: J Dermatol. 2024 Mar 3;51(4):475–83. doi: 10.1111/1346-8138.17096 (PMC11484154; doi:10.1111/1346-8138.17096)
Supplement: Supplementary file 3 — Table S1. [file JDE-51--s004.docx]

**SUPPLEMENTARY TABLE S1** Baseline characteristics and status of avelumab treatment

| **Characteristics** | **N=75** |
| --- | --- |
| Sex, n (%) |  |
| Male | 36 (48.0) |
| Female | 39 (52.0) |
| Age (years) |  |
| Mean (SD) | 77.9 (9.7) |
| Median (range) | 77.0 (42–95) |
| Q1, Q3 | 75.0, 85.0 |
| <65, n (%) | 7 (9.3) |
| 65 to <75, n (%) | 11 (14.7) |
| 75 to <85, n (%) | 37 (49.3) |
| ≥85, n (%) | 20 (26.7) |
| BMI (kg/m^2^) |  |
| Mean (SD) | 23.67 (4.20) |
| Median (range) | 22.51 (14.20–42.80) |
| Q1, Q3 | 20.85, 26.14 |
| <18.5, n (%) | 3 (4.0) |
| 18.5 to <25.0, n (%) | 50 (66.7) |
| 25.0 to <30.0, n (%) | 17 (22.7) |
| 30.0 to <35.0, n (%) | 4 (5.3) |
| 35.0 to <40.0, n (%) | 0 |
| ≥40.0, n (%) | 1 (1.3) |
| Outpatient, n (%) | 22 (29.3) |
| Hospitalized, n (%) | 53 (70.7) |
| Smoking history, n (%) |  |
| Never | 40 (53.3) |
| Previously smoked | 19 (25.3) |
| Sometimes | 1 (1.3) |
| Always | 2 (2.7) |
| Not recorded | 13 (17.3) |
| Drinking history, n (%) |  |
| No | 37 (49.3) |
| Yes | 24 (32.0) |
| Not recorded | 14 (18.7) |
| Pregnant, n (%) | 0 |
| Currently nursing, n (%) | 0 |
| Location of underlying disease, n (%) |  |
| Skin | 67 (89.3) |
| Lymph node | 2 (2.7) |
| Other | 6 (8.0) |
| Duration of disease (months) |  |
| Mean (SD) | 14.99 (17.12) |
| Median (range) | 9.49 (0.2–89.4) |
| Q1, Q3 | 2.73, 20.67 |
| <3 months, n (%) | 20 (26.7) |
| 3 to <12 months, n (%) | 21 (28.0) |
| 12 to <24 months, n (%) | 20 (26.7) |
| ≥24 months, n (%) | 14 (18.7) |
| Lymphatic invasion, n (%) |  |
| No | 20 (26.7) |
| Yes | 51 (68.0) |
| Not recorded | 4 (5.3) |
| Metastasis outside of dermis |  |
| Yes | 60 (80.0) |
| No | 12 (16.0) |
| Not recorded | 3 (4.0) |
| Metastatic lesion, n (%)^†^ |  |
| Bone | 9 (12.0) |
| Muscle | 4 (5.3) |
| Fascia | 1 (1.3) |
| Cartilage tissue | 3 (4.0) |
| Other | 68 (90.7) |
| TNM classification, n (%) |  |
| Tumour |  |
| Tx | 19 (25.3) |
| T0 | 10 (13.3) |
| Tis | 0 |
| T1 | 14 (18.7) |
| T2 | 18 (24.0) |
| T3 | 6 (8.0) |
| T4 | 7 (9.3) |
| Not recorded | 1 (1.3) |
| Lymph nodes |  |
| Nx | 7 (9.3) |
| N0 | 14 (18.7) |
| N1 | 38 (50.7) |
| N2 | 11 (14.7) |
| N3 | 4 (5.3) |
| Not recorded | 1 (1.3) |
| Metastasis |  |
| Mx | 0 |
| M0 | 24 (32.0) |
| M1 | 50 (66.7) |
| Not recorded | 1 (1.3) |
| ECOG PS |  |
| 0 | 36 (48.0) |
| 1 | 25 (33.3) |
| 2 | 7 (9.3) |
| 3 | 6 (8.0) |
| 4 | 0 |
| Unknown | 1 (1.3) |
| PD-L1 status, n (%) |  |
| Negative | 7 (9.3) |
| Positive | 1 (1.3) |
| Unknown | 67 (89.3) |
| Comorbidities, n (%) |  |
| Renal impairment | 11 (14.7) |
| Hepatic impairment | 8 (10.7) |
| ILD | 4 (5.3) |
| Autoimmune disease | 7 (9.3) |
| History of organ transplantation, n (%) | 1 (1.3) |
| Immune suppressant for transplant | 1 (100.0) |
| Treatment history, n (%)^†^ |  |
| Surgery | 57 (76.0) |
| Radiation | 47 (62.7) |
| Chemotherapy | 7 (9.3) |
| Immunotherapy | 0 |
| **Avelumab treatment** |  |
| Reason for avelumab administration, n (%) |  |
| Curatively unresectable MCC | 75 (100.0) |
| Initial dose of avelumab (mg/kg) |  |
| Mean (SD) | 9.9 (0.8) |
| Median (range) | 10.0 (6.0–12.0) |
| Q1, Q3 | 9.9, 10.0 |
| <10, n (%) | 33 (44.0) |
| 10, n (%) | 20 (26.7) |
| >10, n (%) | 22 (29.3) |
| Duration of therapy, n (%) |  |
| ≤6 weeks | 13 (17.3) |
| >6 to ≤12 weeks | 10 (13.3) |
| >12 to ≤24 weeks | 12 (16.0) |
| >24 to ≤36 weeks | 6 (8.0) |
| >36 to ≤52 weeks | 12 (16.0) |
| >52 weeks | 22 (29.3) |
| Number of doses |  |
| Mean (SD) | 12.5 (8.7) |
| Median (range) | 11.0 (1–27) |
| Q1, Q3 | 4.0, 20.0 |
| ≤3, n (%) | 15 (20.0) |
| >3 to ≤6, n (%) | 10 (13.3) |
| >6 to ≤12, n (%) | 15 (20.0) |
| >12 to ≤18, n (%) | 11 (14.7) |
| >18 to ≤26, n (%) | 20 (26.7) |
| >26, n (%) | 4 (5.3) |
| Total dose, mg |  |
| Mean (SD) | 7047.8 (5190.7) |
| Median (range) | 6456.0 (376.0–18750.0) |
| Q1, Q3 | 2350.0, 11400.0 |
| <2350.0 mg, n (%) | 18 (24.0) |
| ≥2350.0 to <6456.0 mg, n (%) | 19 (25.3) |
| ≥6456.0 to <11400.0 mg, n (%) | 19 (25.3) |
| ≥11400.0 mg, n (%) | 19 (25.3) |
| Average dose, mg/kg |  |
| Mean (SD) | 9.775 (0.735) |
| Median (range) | 9.974 (5.88–11.44) |
| Q1, Q3 | 9.587, 10.036 |
| <9.587 mg/kg, n (%) | 19 (25.3) |
| ≥9.587 to <9.974 mg/kg, n (%) | 18 (24.0) |
| ≥9.974 to <10.036 mg/kg, n (%) | 20 (26.7) |
| ≥10.036 mg/kg, n (%) | 18 (24.0) |
| Observation period, weeks |  |
| Mean (SD) | 38.27 (18.88) |
| Median (range) | 51.14 (1.3–61.1) |
| Q1, Q3 | 18.71, 53.00 |
| ≤6 weeks, n (%) | 6 (8.0) |
| >6 to ≤12 weeks, n (%) | 7 (9.3) |
| >12 to ≤24 weeks, n (%) | 7 (9.3) |
| >24 to ≤36 weeks, n (%) | 5 (6.7) |
| >36 to ≤52 weeks, n (%) | 15 (20.0) |
| >52 weeks, n (%) | 35 (46.7) |
| Pre-medication, n (%) | 73 (97.3) |
| Agent given before first avelumab dose |  |
| Acetaminophen + diphenhydramine | 36 (49.3) |
| Acetaminophen + chlorpheniramine maleate | 17 (23.3) |
| Other | 20 (27.4) |
| Concomitant treatment, n (%)^†^ |  |
| Chemotherapy | 1 (1.3) |
| Surgery | 7 (9.3) |
| Radiation therapy | 26 (34.7) |
| Immunotherapy | 0 |
| Abbreviations: BMI, body mass index; ECOG PS, Eastern Cooperative Oncology Group performance status; ILD, interstitial lung disease; MCC, Merkel cell carcinoma; PD-L1, programmed death-ligand 1; Q1, quartile 1; Q3, quartile 3; SD, standard deviation; TNM, tumour, node, metastasis.  ^†^Patients could be counted in more than one category. | |
