# Supplementary material for: Safety and effectiveness of avelumab in patients with Merkel cell carcinoma in general clinical practice in Japan: Post‐marketing surveillance
Source: J Dermatol. 2024 Mar 3;51(4):475–83. doi: 10.1111/1346-8138.17096 (PMC11484154; doi:10.1111/1346-8138.17096)
Supplement: Supplementary file 4 — Table S2. [file JDE-51--s003.docx]

**SUPPLEMENTARY TABLE S2** Adverse drug reactions by system order class, preferred term, and worst grade

| **n (%)** | **Worst Grade** | | | | | **Total** |
| --- | --- | --- | --- | --- | --- | --- |
|  | **1** | **2** | **3** | **4** | **5** |  |
| Patients with ≥1 ADR | 32 (42.7) | 17 (22.7) | 9 (12.0) | 3 (4.0) | 0 | 46 (61.3) |
| Infections and infestations | 1 (1.3) | 0 | 0 | 0 | 0 | 1 (1.3) |
| Nasopharyngitis | 1 (1.3) | 0 | 0 | 0 | 0 | 1 (1.3) |
| Blood and lymphatic system disorders | 0 | 0 | 1 (1.3) | 1 (1.3) | 0 | 1 (1.3) |
| Febrile neutropenia | 0 | 0 | 1 (1.3) | 0 | 0 | 1 (1.3) |
| Thrombocytopenia | 0 | 0 | 0 | 1 (1.3) | 0 | 1 (1.3) |
| Endocrine disorders | 4 (5.3) | 3 (4.0) | 0 | 0 | 0 | 7 (9.3) |
| Adrenal insufficiency | 1 (1.3) | 1 (1.3) | 0 | 0 | 0 | 2 (2.7) |
| Hyperthyroidism | 0 | 1 (1.3) | 0 | 0 | 0 | 1 (1.3) |
| Hypopituitarism | 0 | 1 (1.3) | 0 | 0 | 0 | 1 (1.3) |
| Hypothyroidism | 2 (2.7) | 0 | 0 | 0 | 0 | 2 (2.7) |
| Thyroiditis | 1 (1.3) | 1 (1.3) | 0 | 0 | 0 | 2 (2.7) |
| Metabolism and nutrition disorders | 0 | 2 (2.7) | 2 (2.7) | 0 | 0 | 4 (5.3) |
| Hypokalemia | 0 | 1 (1.3) | 0 | 0 | 0 | 1 (1.3) |
| Hyponatremia | 0 | 1 (1.3) | 1 (1.3) | 0 | 0 | 2 (2.7) |
| Type 1 diabetes mellitus | 0 | 0 | 1 (1.3) | 0 | 0 | 1 (1.3) |
| Nervous system disorders | 0 | 1 (1.3) | 1 (1.3) | 0 | 0 | 2 (2.7) |
| Cerebellar ataxia | 0 | 0 | 1 (1.3) | 0 | 0 | 1 (1.3) |
| Hypoesthesia | 0 | 1 (1.3) | 0 | 0 | 0 | 1 (1.3) |
| Cardiac disorders | 0 | 1 (1.3) | 0 | 0 | 0 | 1 (1.3) |
| Atrial fibrillation | 0 | 1 (1.3) | 0 | 0 | 0 | 1 (1.3) |
| Respiratory, thoracic and mediastinal disorders | 4 (5.3) | 0 | 0 | 0 | 0 | 4 (5.3) |
| Dyspnea | 1 (1.3) | 0 | 0 | 0 | 0 | 1 (1.3) |
| ILD | 2 (2.7) | 0 | 0 | 0 | 0 | 2 (2.7) |
| Respiratory disorder | 1 (1.3) | 0 | 0 | 0 | 0 | 1 (1.3) |
| Gastrointestinal disorders | 4 (5.3) | 2 (2.7) | 0 | 0 | 0 | 5 (6.7) |
| Colitis | 0 | 1 (1.3) | 0 | 0 | 0 | 1 (1.3) |
| Constipation | 1 (1.3) | 0 | 0 | 0 | 0 | 1 (1.3) |
| Diarrhea | 2 (2.7) | 1 (1.3) | 0 | 0 | 0 | 3 (4.0) |
| Vomiting | 1 (1.3) | 0 | 0 | 0 | 0 | 1 (1.3) |
| Hepatobiliary disorders | 1 (1.3) | 0 | 1 (1.3) | 0 | 0 | 2 (2.7) |
| Hepatic function abnormal | 1 (1.3) | 0 | 0 | 0 | 0 | 1 (1.3) |
| Portal vein thrombosis | 0 | 0 | 1 (1.3) | 0 | 0 | 1 (1.3) |
| Skin and subcutaneous tissue disorders | 6 (8.0) | 2 (2.7) | 0 | 0 | 0 | 8 (10.7) |
| Drug eruption | 1 (1.3) | 1 (1.3) | 0 | 0 | 0 | 2 (2.7) |
| Leukoderma | 1 (1.3) | 0 | 0 | 0 | 0 | 1 (1.3) |
| Pruritus | 2 (2.7) | 1 (1.3) | 0 | 0 | 0 | 3 (4.0) |
| Psoriasis | 1 (1.3) | 0 | 0 | 0 | 0 | 1 (1.3) |
| Rash | 1 (1.3) | 0 | 0 | 0 | 0 | 1 (1.3) |
| Musculoskeletal and connective tissue disorders | 1 (1.3) | 1 (1.3) | 1 (1.3) | 0 | 0 | 3 (4.0) |
| Arthritis | 0 | 1 (1.3) | 0 | 0 | 0 | 1 (1.3) |
| Myositis | 0 | 0 | 1 (1.3) | 0 | 0 | 1 (1.3) |
| Pain in extremity | 1 (1.3) | 0 | 0 | 0 | 0 | 1 (1.3) |
| Renal and urinary disorders | 0 | 1 (1.3) | 0 | 0 | 0 | 1 (1.3) |
| Renal disorder | 0 | 1 (1.3) | 0 | 0 | 0 | 1 (1.3) |
| General disorders and administration site conditions | 14 (18.7) | 2 (2.7) | 1 (1.3) | 0 | 0 | 17 (22.7) |
| Asthenia | 1 (1.3) | 0 | 0 | 0 | 0 | 1 (1.3) |
| Chills | 5 (6.7) | 0 | 0 | 0 | 0 | 5 (6.7) |
| Gait disturbance | 1 (1.3) | 0 | 0 | 0 | 0 | 1 (1.3) |
| Malaise | 3 (4.0) | 0 | 0 | 0 | 0 | 3 (4.0) |
| Pyrexia | 11 (14.7) | 2 (2.7) | 1 (1.3) | 0 | 0 | 14 (18.7) |
| Investigations | 6 (8.0) | 2 (2.7) | 1 (1.3) | 0 | 0 | 9 (12.0) |
| Alanine aminotransferase increased | 2 (2.7) | 0 | 0 | 0 | 0 | 2 (2.7) |
| Aspartate aminotransferase increased | 2 (2.7) | 0 | 0 | 0 | 0 | 2 (2.7) |
| Blood thyroid stimulating hormone abnormal | 1 (1.3) | 0 | 0 | 0 | 0 | 1 (1.3) |
| Blood thyroid stimulating hormone decreased | 1 (1.3) | 0 | 0 | 0 | 0 | 1 (1.3) |
| Increased C-reactive protein | 1 (1.3) | 0 | 0 | 0 | 0 | 1 (1.3) |
| Eosinophil count increased | 1 (1.3) | 0 | 0 | 0 | 0 | 1 (1.3) |
| Gamma-glutamyl transferase increased | 1 (1.3) | 0 | 0 | 0 | 0 | 1 (1.3) |
| Neutrophil count decreased | 0 | 0 | 1 (1.3) | 0 | 0 | 1 (1.3) |
| Thyroid function test abnormal | 0 | 1 (1.3) | 0 | 0 | 0 | 1 (1.3) |
| White blood cell count decreased | 0 | 0 | 1 (1.3) | 0 | 0 | 1 (1.3) |
| Computerized tomogram thorax abnormal | 1 (1.3) | 0 | 0 | 0 | 0 | 1 (1.3) |
| Hepatic enzyme increased | 0 | 1 (1.3) | 0 | 0 | 0 | 1 (1.3) |
| Procalcitonin increased | 1 (1.3) | 0 (0.00) | 0 | 0 | 0 | 1 (1.3) |
| Injury, poisoning and procedural complications | 3 (4.0) | 3 (4.0) | 1 (1.3) | 2 (2.7) | 0 | 9 (12.0) |
| Radiation pneumonitis | 0 | 0 | 0 | 1 (1.3) | 0 | 1 (1.3) |
| Infusion related reaction | 3 (4.0) | 3 (4.0) | 1 (1.3) | 1 (1.3) | 0 | 8 (10.7) |
| Patients receiving corticosteroids for an ADR | 0 | 3 (4.0) | 3 (4.0) | 2 (2.7) | 0 | 8 (10.7) |
| Abbreviations: ADR, adverse drug reaction; ILD, interstitial lung disease. | | | | | | |
