# Supplementary material for: Safety and effectiveness of avelumab in patients with Merkel cell carcinoma in general clinical practice in Japan: Post‐marketing surveillance
Source: J Dermatol. 2024 Mar 3;51(4):475–83. doi: 10.1111/1346-8138.17096 (PMC11484154; doi:10.1111/1346-8138.17096)
Supplement: Supplementary file 5 — Table S3. [file JDE-51--s009.docx]

**SUPPLEMENTARY TABLE S3** Incidence of adverse drug reactions of safety specifications

| **n (%)** | **Safety analysis set (N=75)** | | |
| --- | --- | --- | --- |
|  | **Non-serious** | **Serious** | **Total** |
| Infusion reactions^†^ | 17 (22.7) | 5 (6.7) | 21 (28.0) |
| Thyroid dysfunction | 5 (6.7) | 2 (2.7) | 7 (9.3) |
| Hepatic function disorders | 4 (5.3) | 0 | 4 (5.3) |
| ILD | 2 (2.7) | 1 (1.3) | 3 (4.0) |
| Nerve disorders | 1 (1.3) | 1 (1.3) | 2 (2.7) |
| Adrenal insufficiency | 2 (2.7) | 0 | 2 (2.7) |
| Colitis/severe diarrhea | 1 (1.3) | 0 | 1 (1.3) |
| Type 1 diabetes | 0 | 1 (1.3) | 1 (1.3) |
| Myositis/rhabdomyolysis | 0 | 1 (1.3) | 1 (1.3) |
| Renal disorders | 1 (1.3) | 0 | 1 (1.3) |
| Myocarditis | 0 | 0 | 0 |
| Encephalitis/meningitis | 0 | 0 | 0 |
| Embryo and fetal toxicity | – | – | – |
| Transplant rejection or graft-versus-host disease in patients with organ transplant history^‡^ | 0 | 0 | 0 |
| Abbreviations: ADR, adverse drug reaction; ILD, interstitial lung disease.  ^†^One patient developed both serious and non-serious ADRs.  Serious ADR: Any untoward medical occurrence that at any dose: results in death, is life threatening, requires inpatient hospitalization or prolongation of existing hospitalization, results in persistent or significant disability/incapacity, or is a congenital anomaly/birth defect.  ^‡^One patient had a history of organ transplantation. | | | |
