# Supplementary material for: Safety and effectiveness of avelumab in patients with Merkel cell carcinoma in general clinical practice in Japan: Post‐marketing surveillance
Source: J Dermatol. 2024 Mar 3;51(4):475–83. doi: 10.1111/1346-8138.17096 (PMC11484154; doi:10.1111/1346-8138.17096)
Supplement: Supplementary file 6 — Table S4. [file JDE-51--s002.docx]

**SUPPLEMENTARY TABLE S4** Detailed timing of patient’s first experiences of infusion reactions

| **Avelumab dose** | **Timing of infusion reaction onset** | | | | | **Total number of patients** |
| --- | --- | --- | --- | --- | --- | --- |
|  | **During administration,**  **n (%)** | **Immediately after the start of administration,**  **n (%)** | **Within 1 hour after the end of administration,**  **n (%)** | **At least 1 hour after the end of infusion, n (%)** | **Unknown, n (%)** |  |
| 1st | 11 (57.9) | 0 | 2 (10.5) | 6 (31.6) | 0 | 19 |
| 2nd | 0 | 0 | 0 | 0 | 0 | 0 |
| 3rd | 0 | 1 (100.0) | 0 | 0 | 0 | 1 |
| 4th | 0 | 0 | 0 | 0 | 0 | 0 |
| 5th | 0 | 0 | 0 | 0 | 0 | 0 |
| 6th | 0 | 0 | 0 | 0 | 0 | 0 |
| 7th | 0 | 0 | 0 | 0 | 1 (100.0) | 1 |
| Total | 11 (52.4) | 1 (4.8) | 2 (9.5) | 6 (28.6) | 1 (4.8) | 21 |
| No infusion reaction was observed after the 8^th^ avelumab dose in any patient. Incidence rates (%) were calculated by dividing the actual number of infusion reactions by the total number of patients in each group. | | | | | | |
