# Supplementary material for: Safety and effectiveness of avelumab in patients with Merkel cell carcinoma in general clinical practice in Japan: Post‐marketing surveillance
Source: J Dermatol. 2024 Mar 3;51(4):475–83. doi: 10.1111/1346-8138.17096 (PMC11484154; doi:10.1111/1346-8138.17096)
Supplement: Supplementary file 7 — Table S5. [file JDE-51--s006.docx]

**SUPPLEMENTARY TABLE S5** Pre-medication administered at the first dose of avelumab

| **Pre-medication at first dose of avelumab** | **Safety analysis set (N=75)** | **Patients with infusion reaction, n (%)** |
| --- | --- | --- |
| No pre-medication | 2 | 0 |
| Pre-medication administered | 73 | 21 (28.8) |
| Acetaminophen and diphenhydramine | 36 | 9 (25.0) |
| Acetaminophen and chlorpheniramine maleate^†^ | 17 | 6 (35.3) |
| Others | 20 | 6 (30.0) |
| ^†^In combination with famotidine (n=2). | | |
