# Supplementary material for: Safety and effectiveness of avelumab in patients with Merkel cell carcinoma in general clinical practice in Japan: Post‐marketing surveillance
Source: J Dermatol. 2024 Mar 3;51(4):475–83. doi: 10.1111/1346-8138.17096 (PMC11484154; doi:10.1111/1346-8138.17096)
Supplement: Supplementary file 8 — Table S6. [file JDE-51--s007.docx]

**SUPPLEMENTARY TABLE S6** Best overall response (within 52 weeks after the first administration of avelumab)

| **n (%)** | **Avelumab (N=75)** |
| --- | --- |
| ORR (CR + PR) | 34 (45.3) |
| (95% CI) | (33.8 to 57.3) |
| DCR (CR + PR + SD) | 42 (56.0) |
| (95% CI) | (44.1 to 67.5) |
| CR | 18 (24.0) |
| PR | 16 (21.3) |
| SD | 8 (10.7) |
| PD | 25 (33.3) |
| Unknown | 8 (10.7) |
| Abbreviation: CI, confidence interval; CR, complete response; DCR, disease control rate; NE, not evaluable; ORR, objective response rate; PD, progressive disease; PR, partial response; SD; stable disease. | |
