# Supplementary material for: Safety and effectiveness of avelumab in patients with Merkel cell carcinoma in general clinical practice in Japan: Post‐marketing surveillance
Source: J Dermatol. 2024 Mar 3;51(4):475–83. doi: 10.1111/1346-8138.17096 (PMC11484154; doi:10.1111/1346-8138.17096)
Supplement: Supplementary file 9 — Data S1. [file JDE-51--s008.docx]

**Supplementary Methods**

**Definition of infusion reactions**

The definition of infusion reactions in this PMS followed the definitions used by the JAVELIN Merkel 200 study.^1^ The following MedDRA Preferred Terms ([PT] per MedDRA/J version 24.0), whose onset and recovery time met the following criteria, were considered avelumab-related infusion reactions.

1. **Diagnosis-related infusion reactions:** infusion related reaction, drug hypersensitivity, anaphylactic reaction, hypersensitivity, and type I hypersensitivity occurring on or the day after drug administration.
2. **Symptom-related infusion reactions:** pyrexia, chills, flushing, hypotension, dyspnea, wheezing, back pain, abdominal pain, and urticaria occurring on the day of drug administration and resolving/being fully resolved within 3 days from onset.

Events reported as an infusion reaction by investigators were also aggregated as infusion reactions after the sponsor performed a separate medical assessment.

**References**

1. Kaufman HL, Russell J, Hamid O, Bhatia S, Terheyden P, D'Angelo SP, et al. Avelumab in patients with chemotherapy-refractory metastatic Merkel cell carcinoma: a multicentre, single-group, open-label, phase 2 trial. Lancet Oncol. 2016;17:1374-85.
